# A Highly Efficient CRISPR-Cas9-Mediated Large Genomic Deletion in *Bacillus subtilis*

**DOI:** 10.3389/fmicb.2017.01167

**Published:** 2017-06-23

**Authors:** Younju So, Soo-Young Park, Eun-Hye Park, Seung-Hwan Park, Eui-Joong Kim, Jae-Gu Pan, Soo-Keun Choi

**Affiliations:** ^1^Infectious Disease Research Center, Korea Research Institute of Bioscience and BiotechnologyDaejeon, South Korea; ^2^Department of Biosystems and Bioengineering, KRIBB School of Biotechnology, Korea University of Science and Technology (UST)Daejeon, South Korea; ^3^Genofocus Inc.Daejeon, South Korea

**Keywords:** CRISPR-Cas9, *Bacillus subtilis*, genome engineering, large genomic deletion, genomic point mutation, gene insertion

## Abstract

In *Bacillus subtilis*, large genomic deletions have been carried out for genome reduction, antibiotic overproduction, and heterologous protein overexpression. In view of the eco-friendliness of *B. subtilis*, it is critical that engineering preserves its food-grade status and avoids leaving foreign DNA in the genome. Existing methods of generating large genomic deletions leave antibiotic resistance markers or display low mutation efficiency. In this study, we introduced a clustered regularly interspaced short palindromic repeat-derived genome engineering technique to develop a highly efficient method of generating large genomic deletions in *B. subtilis* without any trace of foreign DNA. Using our system, we produced 38 kb plipastatin-synthesizing *pps* operon deletion with 80% efficiency. The significant increase in mutation efficiency was due to plasmids-delivered *Streptococcus pyogenes*-originated SpCas9, target-specific sgRNA and a donor DNA template, which produces SpCas9/sgRNA endonuclease complex continuously for attacking target chromosome until the mutagenic repair occurs. Our system produced single-gene deletion in *spo0A* (∼100%), point mutation (∼68%) and GFP gene insertion (∼97%) in *sigE* and demonstrated its broad applicability for various types of site-directed mutagenesis in *B. subtilis*.

## Introduction

Previous studies have shown that large genomic deletions in *Bacillus* sp. play an important role for heterologous enzyme expression, genome reduction (Westers et al., 2003), stain improvement (Thwaite et al., 2002), and overproduction of antibiotics (Zobel et al., 2015). The propionly-CoA-utilizing *prpBD* operon deletion strain, *Bacillus subtilis* JK125, showed a 75% increase in production of *Saccharopolyspora erythraea* derived 6-deoxyerythronolide B relative to the wild type strain *B. subtilis* JK120 (Kumpfmuller et al., 2016). To determine non-essential region for *B. subtilis* cell growth, 7.7% of the 4.2 Mb was deleted including three large gene clusters *pks*, *pps*, and *srf* (Westers et al., 2003). To increase the stability of recombinant anthrax protective antigen (rPA) secretion, the *dltABCDE* operon was inactivated by single cross-over integration of a pMUTIN4 containing erythromycin resistance marker. Deletion of the *dlt* operon inhibited the D-alanylation of teichoic acids, and resulted in increased resistance to proteolysis (Thwaite et al., 2002). A large *pks* operon was removed to investigate the heterologous expression of *esyn* cluster that produce an antibiotic Enniatin in *B. subtilis* (Zobel et al., 2015). *B. subtilis* is one of the main industrial enzyme producers and a GRAS (generally regarded as safe) microbial host. Unfortunately, the conventional methods for generating large genomic deletions rely on insertion of an antibiotic resistance marker and disqualifies an engineered *Bacillus* strain from use as an eco-friendly host for food-grade applications, industrial fermentation, and bioremediation.

Counter-selectable markers such as *upp* and *araR* have been used in *B. subtilis* to avoid insertion of an antibiotic resistance marker at the desired mutation region (Dong and Zhang, 2014). Using *upp*, 0.87 Mb of *B. subtilis* genome including two large operons *pks* and *pps* were removed. The resulting MGB874 strain displayed increased extracellular cellulase and protease productivity (Morimoto et al., 2008). The arabinose operon repressor (*araR*) has been used to generate 3.8 kb (*iolS*-*csbC*) and 41.8 kb (*hutM*-*csbC*) deletion mutant in *B. subtilis* precisely (Liu et al., 2008). Although these counter-selectable markers successfully removed the target gene without using antibiotics resistance markers, the system used previously constructed *upp*- and *araR*- inactivated strains (*upp*::*erm* and *araR*::*neo*) that left traces of foreign DNA on the secondary region of the chromosome. Synthetic gene circuit, a recently developed counter-selectable marker system, was able to produce plipastatin synthetase (*pps*) operon deletion without leaving any trace of foreign DNA on the chromosome. However, the mutation efficiency was low as 6.4% (Jeong et al., 2015). This emphasizes the necessity for a highly efficient method of generating large genomic deletions without leaving any trace of foreign DNA on the genome.

A clustered regularly interspaced short palindromic repeat (CRISPR) system, which uses an RNA-guided DNA endonuclease was first known as a bacterial adaptive immune response and was inevitably developed into a genome engineering technique (Deltcheva et al., 2011; van der Oost et al., 2014). *Streptococcus pyogenes*-originated Cas9 (SpCas9) is widely used for a CRISPR-mediated genome engineering and requires sgRNA containing 20 bp gRNA sequence, which is complementary to the target region where double-strand break (DSB) should occurs, as well as the SpCas9 binding scaffold. After recognizing protospacer adjacent motif (PAM) located at 3′-end of the 20 bp gRNA sequence, sgRNA-SpCas9 endonuclease complex generates the DSB at 20 bp-designated target region (Brouns et al., 2008; Garneau et al., 2010). Two types of machinery can be used to repair the breakage: homology directed repair (HDR) and non-homologous end joining (NHEJ). During HDR, a donor DNA template containing the desired mutation is introduced into the host cell to repair the DSB. For HDR, the donor DNA template should contain a region homologous to the chromosome along with the desired mutation site to recover the DSB via recombination. With NHEJ, break ends are directly repaired by ligase activity while InDels occur without insertion of the donor DNA (Ran et al., 2013).

The CRISPR-Cas9-mediated genome engineering method overcame the limitations that the previous methods had, and started to generate reliable foreign DNA-free mutants in many wild type bacterial strains, including *B. subtilis*. First CRISPR-Cas9-mediated prokaryotic genome engineering was introduced with *S. pneumoniae* and *Escherichia coli* (Jiang et al., 2013). In *E. coli*, single-stranded DNA (ssDNA)-mediated HDR was used for point mutation of the *rpsL* gene with a 65% mutation efficiency. Improved mutation efficiencies using CRISPR-Cas9 system were obtained after secondary engineering methods, such as λ-Red recombineering system, were supplemented (Mougiakos et al., 2016). Even though the CRISPR technology has been applied to many different microorganisms including *Lactobacillus reuteri* (Oh and van Pijkeren, 2014) and *Streptomyces coelicolor* (Huang et al., 2015), the reports on using CRISPR-Cas9 system for *Bacillus* genome engineering are scarce. Using CRISPR-Cas9, multiple genes including *srfC, spoIIAC, nprE, aprE*, and *amyE* were manipulated to obtain β-CGTase overexpressed antifoaming *B. subtilis* ATCC 6051a (Zhang et al., 2016). The authors attempted to generate a 284 bp region deletion in the *srfC* gene and reported that the deletion mutation efficiency was low as 9.1%. The efficiency of further serial knockout mutations in *spoIIAC, nprE, aprE*, and *amyE* ranged from 33 to 55%.

Because of the low mutation efficiency, CRISPR-Cas9-mediated *B. subtilis* genome engineering still requires the use of counter-selectable markers to increase the mutation efficiency (Westbrook et al., 2016). Moreover, the application of large genomic deletion using CRISPR-Cas9 is still unknown in *B. subtilis*. Therefore, we developed a highly efficient genome engineering system based on CRISPR-Cas9, and generated a precise large genomic deletion in *B. subtilis.* The system uses plasmids to deliver sgRNA transcription module, donor DNA template and SpCas9, and achieves the large genomic deletion with 80% efficiency. As well as generating large genomic deletion, the system also proved it as an efficient genome engineering method by generating base deletion (∼100%), point mutation (∼68%) and gene insertion (∼97%) with high efficiency.

## Materials and Methods

### Strains and Culture Conditions

The *B. subtilis* strains and plasmids used in this study are listed in **Table 1**. *E. coli* MC1061 was used to construct recombinant plasmids. *B. subtilis* cells were cultured in Luria–Bertani (LB) broth or LB agar (Difco, Detroit, MI, United States) media at 37°C for screening and flow cytometry measurement. When required, the antibiotics were supplemented with following conditions for selection purposes: ampicillin (100 μg/ml), chloramphenicol (5 μg/ml), or neomycin (10 μg/ml). Transformation of *B. subtilis* cells was carried out by the two-step transformation procedure (Cutting and Harwood, 1990).

**Table 1:** *Bacillus* strains and plasmids used in this study.

Strain/plasmid	Genotype/description	Reference
***Bacillus* strains**		
*B. subtilis* 168	Tryptophan auxotrophic (trpC2)	Laboratory stock
BS-C100	*Bacillus subtilis* 168 carrying the plasmid pHCas9	This study
BS-C101	Strain BS-C100 carrying the plasmid pB0A-2	This study
BS-C102	Strain BS-C100 carrying the plasmid pB0A-1	This study
BS-C103	Strain BS-C100 carrying the plasmid pB0A-800	This study
BS-C104	Strain BS-C100 carrying the plasmid pB0A-400	This study
BS-C105	Strain BS-C100 carrying the plasmid pB0A-200	This study
BS-C106	Strain BS-C100 carrying the plasmid pB0A-100	This study
BS-C107	Strain BS-C100 carrying the plasmid pB0A-d500	This study
BS-C108	Strain BS-C100 carrying the plasmid pB0A-d1000	This study
BS-C109	Strain BS-C100 carrying the plasmid pBP-dpps	This study
BS-C110	Strain BS-C100 carrying the plasmid pBE-pm	This study
BS-C111	Strain BS-C100 carrying the plasmid pBE-gfp	This study
**Plasmids**		
pAD123	*E. coli*–*Bacillus* shuttle plasmid, promoter-less *gfpmut3*, chloramphenicol resistance in *Bacillus*	BGSC
pHT01	*E. coli*–*Bacillus* shuttle plasmid carrying P*grac* and *lacI*	MoBiTec
pHCas9	pHT01 derivative plasmid, containing P*grac*-SpCas9	This study
pB0A-2	pAD123 derivative, containing *spo0A* targeting 20 bp gRNA transcription module and 2 kb donor DNA	This study
pB0A-1	pAD123 derivative, containing *spo0A* targeting 20 bp gRNA transcription module and 1 kb donor DNA	This study
pB0A-800	pAD123 derivative, containing *spo0A* targeting 20 bp gRNA transcription module and 800 bp donor DNA	This study
pB0A-400	pAD123 derivative, containing *spo0A* targeting 20 bp gRNA transcription module and 400 bp donor DNA	This study
pB0A-200	pAD123 derivative, containing *spo0A* targeting 20 bp gRNA transcription module and 200 bp donor DNA	This study
pB0A-100	pAD123 derivative, containing *spo0A* targeting 20 bp gRNA transcription module and 100 bp donor DNA	This study
pB0A-d500	pAD123 derivative, containing *spo0A* targeting 20 bp gRNA transcription module for generating *spo0A* 500 bp deletion	This study
pB0A-d1000	pAD123 derivative, containing *spo0A* targeting 20 bp gRNA transcription module for generating *spo0A* 1 kb deletion	This study
pBE-pm	pAD123 derivative, generating *sigE* point mutation	This study
pBE-gfp	pAD123 derivative, generating *gfp* insertion at *sigE*	This study

### Plasmids Construction

#### Construction of pHCas9

**FIGURE 1:**
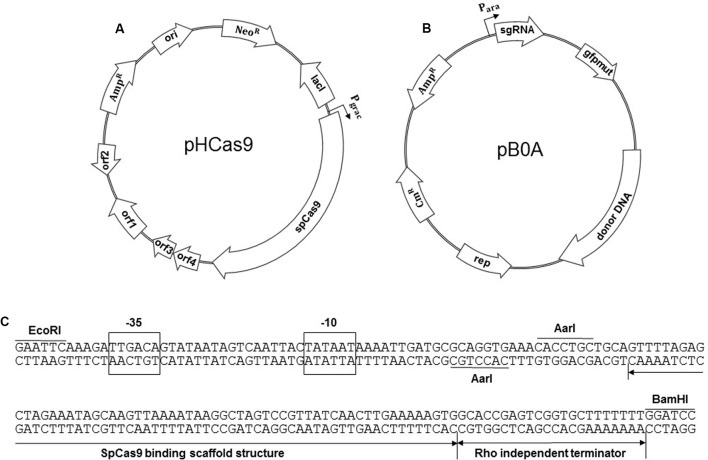
Construction of the two plasmids for an iterative genome engineering system. **(A)** Maps of pHCas9 plasmid and **(B)** pB0A plasmid containing *spo0A* sgRNA transcription module as well as donor DNA template. **(C)** Sequence of a synthetic sgRNA module containing *araABCD* promoter, AarI sites for cloning of 20 bp gRNA sequence, SpCas9 binding scaffold and transcriptional terminator. The plasmids were deposited at Korean Collection for Type Cultures (KCTC) as KCTC 11628 (pHCas9) and KCTC 11629 (pB0A).

The pHT-*neo* plasmid was derived from pHT01, an expression plasmid for *B. subtilis* (Mobitech, Gottingen, Germany). The chloramphenicol-resistant gene (*cat*) of pHT01 was replaced with a neomycin-resistance gene (*neo*) by amplifying the *neo* gene from pUB110 (Keggins et al., 1978) using the primers neo-F and neo-R. The PCR fragment was ligated to SphI- and NheI-treated pHT01 to produce pHT-*neo*. BglII- and AatII-digested SpCas9 gene from pwtCas9-bacteria (Addgene, Cambridge, MA, United States) was further ligated with BamHI- and AatII-digested pHT-*neo* to construct a plasmid pHCas9 (**Figure 1A**).

#### Construction of pB0A

**FIGURE 2:**
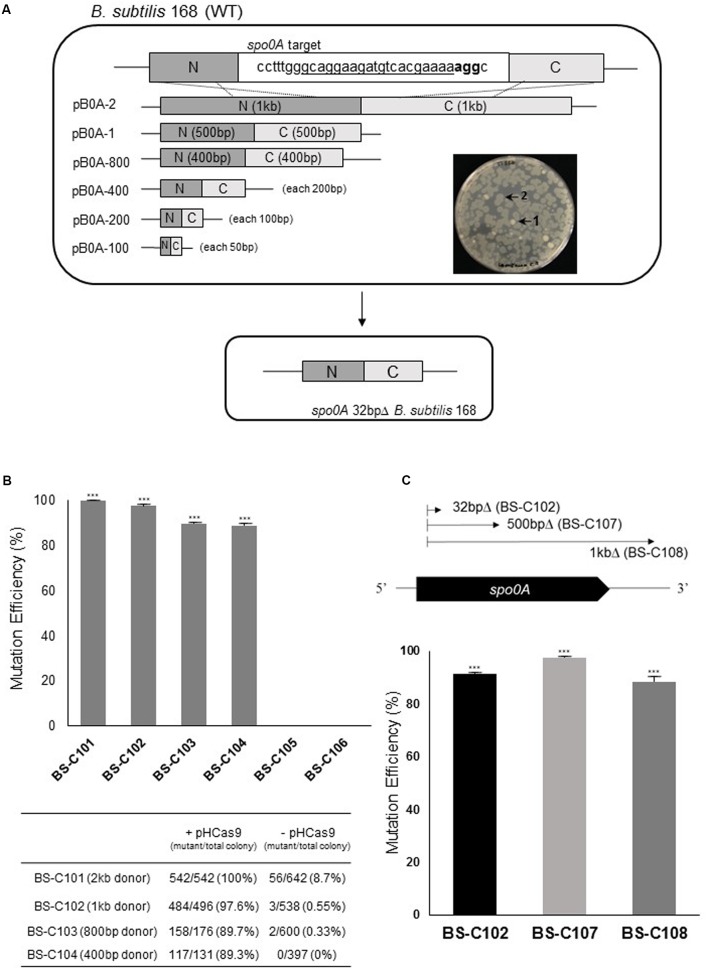
Donor DNA size-dependent HDR in *Bacillus subtilis* using CRISPR-Cas9. **(A)** 32 of 804 bp in *B. subtilis spo0A* were deleted by six different donor DNA templates using homologous recombination. The underlined 20 bp sequence was given as gRNA. Nucleotides shown in bold (agg) are the protospacer adjacent motif (PAM) region. SpCas9-mediated DSB occurred between the 17th adenine (a) and 18th adenine (a) in the gRNA sequence. Significant morphological change (transparent or clear) was observed with *spo0A* mutant (2) compare to the wild type *B. subtilis* 168 (1). **(B)** Mutation efficiencies based on different sizes of donor DNA templates: 2 kb (pB0A-2) to 100 bp (pB0A-100). Means of three independent technical replica experiments are presented; the error bars indicate standard deviations. ^∗∗∗^*P* < 0.001 compared with control without presence of pHCas9. **(C)** Correlation between mutation efficiency and the size of the deletion region. The HDR-mediated two-plasmids CRISPR-Cas9 system was efficient in generating 32 bp (BS-C102), 500 bp (BS-C107) and 1 kb (BS-C108) deletions in *spo0A*. Means of three independent technical replica experiments are presented; the error bars indicate standard deviations. ^∗∗∗^*P* < 0.001 compared with control without presence of pHCas9.

To generate the *spo0A* deletion mutant, oligonucleotides for 20 bp gRNA (spo0A-F and spo0A-R) were synthesized and ligated to an AarI-digested pAgR. *spo0A*-targeting gRNA containing pAgR was named p0AgR (**Figure 1B**). The -10 to -35 region of *sigA*-dependent *araABCD* promoter of *B. subtilis* was used to produce synthetic sgRNA including a SpCas9 binding scaffold. Two AarI restriction enzyme sites were designed for precise cloning of the downstream transcription start site (TSS) and a 20 bp target guide RNA (gRNA) sequence (**Figure 1C**). The EcoRI- and BamHI-digested synthetic sgRNA module and pAD123 (Dunn and Handelsman, 1999) produced pAgR. We designed various sizes of DNA templates (2 kb to 100 bp) for proper HDR, to generate a 32 bp deletion in *spo0A* (**Figure 2A**). A 2 kb donor DNA template, containing the 1 kb N-terminus and 1 kb C-terminus from the mutation site, was PCR-amplified using the *B. subtilis* 168 chromosome as a template. Primer sets 0AT-F1/0AT-R1 and 0AT-F2/0AT-R2 were used to amplify the 2 kb donor DNA template. The 2 kb donor DNA template was further digested with SpeI and SalI for ligation with p0AgR. Ligation of the 2 kb donor DNA and p0AgR constructed pB0A-2. The 2 kb donor DNA template was further truncated to 1 kb (500 bp each from the N- and C-termini) by PCR using primers 0AT-F500 and 0AT-R500. Smaller donor DNA templates (800, 400, 200, and 100 bp) were PCR-amplified using appropriate primers listed in **Table 2**. The PCR-amplified donor DNA templates were subsequently digested with SpeI and SalI for ligation with the *spo0A* sgRNA-containing plasmid p0AgR to produce the following plasmids: pB0A-1, pB0A-800, pB0A-400, pB0A-200, and pB0A-100.

**Table 2:** Oligonucleotides and primers used in this study.

Oligonucleotide	Sequence (5′ → 3′)
**spo0A-F**	attg**gcaggaagatgtcacgaaaa**
**spo0A-R**	aaac**ttttcgtgacatcttcctgc**
**sigE-PMF**	attg**gatgaagtctattacatagg**
**sigE-PMR**	aaac**cctatgtaatagacttcatc**
**sigE-GIF**	attg**gatgaagtctattacatagg**
**sigE-GIR**	aaac**cctatgtaatagacttcatc**
**ppsA-F**	attg**tatcctcttattatgagaac**
**ppsA-R**	aaac**gttctcataataagaggata**
**ppsE-F**	attg**gctcataaagacatgctgga**
**ppsE-R**	aaac**tccagcatgtctttatgagc**

**Primer**	**Sequence (5′ → 3′)**

**neo-F**	atagcatgctgatgacacagaagaaggcg
**neo-R**	atagctagcgcaatgccgggatagac
**0AT-F1**	atgagctcactagttcaattgaaaaagggacagg
**0AT-R1**	yctgtcagcataatgacattc
**0AT-F2**	tcattatgctgacaggtcgatttaggcgcgtccta
**0AT-R2**	atatgcatgtcgaccgctatggcaagcaattgtg
**0AT-F500**	ataactagtcagtttttttattttgatccctct
**0AT1-R500**	aatgtcgactgctctaacctcagcttatcc
**0AT-F200**	attactagtctgttaagtgaatatataga
**0AT-R200**	atgtcgacgatagagatagcctttaata
**0AT-F100**	ttaactagtcttgcggttttagagaggct
**0AT-R100**	atagtcgacatttttcttctttggttcag
**0AT-F50**	attactagtgctgagggaatcagatctgaaaaaacagccgaatgtcatt atgctgacag
**0AT-R50**	atgtcgacttttccatatcaaacggtttgagaataaagtaggacgcgcct aaatcgac
**0A500-N-F1**	tatactagttaaatccactgtaacatcaa
**0A500-N-R1**	tgctgcgtataatactgctttgcttttgtatattttaccgtat
**0A500-C-F1**	gcaaagcagtattatacgcagca
**0A500-C-R1**	atagtcgacatttgctttccttacttttc
**0A1kb-N-F1**	atactagtttaacggttattgatgatga
**0A1kb-N-R1**	acgattttgcaggagaaaggaaatgtagttaacaggattc
**0A1kb-C-F1**	cctttctcctgcaaaatcgt
**0A1kb-C-R1**	attatgcatgtcgacatcttcatatctcactgat
**sigE-NF1**	atactagtacacttcatgtcagaggcct
**sigE-NR1**	gcttcactcccgcctatgtactagacttcatcacttttca
**sigE-CF1**	tgaaaagtgatgaagtctagtacataggcgggagtgaagc
**sigE-CR1**	atgtcgaccaagcccaaaccgcagctcca
**sigE-NR2**	acttttcagcccaagtttca
**sigE-CF2**	cgggagtgaagccctgccgc
**sigE-Pxyl-F1**	cttgggctgaaaagtacattgaaataaacatttat
**sigE-Pxly-R2**	ttgtcatttccccctttgat
**Pxyl-gfp-F1**	agggggaaatgacaaagtaaaggagaagaactttt
**Pxyl-gfp-R1**	agggcttcactcccgttatttgtatagttcatcca
**ppsEsg-F1**	attagatctaagcttaaagattgacagtataatag
**ppsEsg-R1**	aatactagtaatgcggccgcaatggatccaaaaaaagcaccgac tcggtgccac
**pps-F1**	atactagtaggaaaagtaagagcaaaaa
**pps-NR1**	gaaaaaagcagaaaaatgac
**pps-CF1**	gtcatttttctgcttttttctaaagcggattagcggacag
**pps-R1**	atgtcgacgaaccgcttttaggacttt
**pps-R2**	gaatgcctggatgataata
**ppsA-F1**	tgttttagatccgcatttagc
**ppsA-R1**	tcgttcctgacgtataaatg
**ppsB-F1**	ggcattaagcgtggagagtg
**ppsB-R1**	gccgttaccccttttaccaa
**ppsC-F1**	tgatgccgcagcaacctgaa
**ppsC-R1**	gtgctcaacggccactcctt
**ppsE-F1**	cactaatgaatccgtgaaga
**ppsE-R1**	tttcgttaagcctgtatgcc

*^∗^Underlined sequences are the restriction enzyme sites. ^∗∗^Bolded sequences represent the 20 bp synthetic gRNA.*

To generate a 500 bp deletion in *spo0A*, we constructed pB0A-d500. 500 bp of each N-terminus and C-terminus from the mutation site were PCR-amplified using primer sets 0A500-N-F1/0A500-N-R1 and 0A500-C-F1/0A500-C-R1, and the *B. subtilis* 168 chromosome was used for the PCR template. Then we fused the resulting PCR amplicons and digested with SpeI and SalI for ligation with p0AgR. To construct pB0A-d1000 for generating 1 kb deletion in *spo0A* gene, primer sets 0A1kb-N-F1/0A1kb-N-R1 and 0A1kb-C-F1/0A1kb-C-R1 were used to amplify the N-terminus and C-terminus of the donor DNA template. Subsequent fusion PCR produced an N- and C-termini-fused 1 kb-sized donor DNA template. We digested both donor DNA template and p0AgR with SpeI and SalI and the ligation created pB0A-d1000.

#### Construction of pBP-dpps

For *pps* operon deletion, *ppsA* gRNA-containing oligonucleotides (ppsA-F and ppsA-R) and *ppsE* gRNA-containing oligonucleotides (ppsE-F and ppsE-R) were synthesized and each ligated to an AarI-digested pAgR. The *ppsE*-targeting sgRNA transcription module was amplified using primers ppsEsg-F1 and ppsEsg-R1, and digested with BglII and SpeI. Digested *ppsE* sgRNA-containing PCR amplicon was further ligated to a BamHI- and SpeI-digested *ppsA*-targeting sgRNA transcription module containing plasmid, to produce pPgR. To generate a donor DNA template for *pps* operon deletion, the N- and C-termini of the *pps* operon were amplified using primer sets pps-F1/pps-NR1 and pps-CF1/pps-R1. Fusion PCR using primers pps-F1 and pps-R1 generated a 1 kb donor DNA template for *pps* operon deletion. A pPgR, which contained both *ppsA* and *ppsE*-targeting sgRNA transcription modules was digested with SpeI and SalI. Further ligation with a 1 kb donor DNA template produced pBP-dpps.

#### Construction of pBE-pm

To construct pBE-pm, *sigE* gRNA-containing oligonucleotides (sigE-PMF and sigE-PMR) were synthesized and ligated to an AarI-digested pAgR. A 1 kb donor DNA template containing a *sigE* point mutation (T → A) was amplified and fused using primer sets sigE-NF1/sigE-NR1 and sigE-CF1/sigE-CR1. The donor DNA template was digested with SpeI and SalI for ligation with pEgR, containing a *sigE* sgRNA transcribing module.

#### Construction of pBE-gfp

pBE-gfp was constructed to demonstrate insertion of a green fluorescence protein (GFP) into the *sigE* gene. First, *sigE* gRNA-containing oligonucleotides (sigE-GIF and sigE-GIR) were ligated to pAgR. Subsequently, the N- and C-termini of the *sigE* target region were PCR-amplified using primer sets sigE-NF1/sigE-NR2 and sigE-CF2/sigE-CR1. P_xyl_ promoter from pUlacI-nprE (Jeong et al., 2015) was amplified using sigE-Pxyl-F1 and sigE-Pxly-R2. The amplicon was further fused with the N-terminus of the *sigE* gene, using primers sigE-NF1 and sigE-Pxyl-R2. The GFP gene was amplified from pAD123 using primers Pxyl-gfp-F1 and Pxyl-gfp-R1. Then the amplicon was fused with the C-terminus of the *sigE* gene with primers Pxyl-gfp-F1 and sigE-CR1. Final fusion PCR with primers sigE-NF1 and sigE-CR1 created a 1.8 kb *sigE* donor DNA template containing the P_xyl_ promoter and the GFP gene. This PCR product was digested with SpeI and SalI for ligation with pEgR, produced pBE-gfp.

### CRISPR-Cas9-Mediated Mutagenesis of *B. subtilis*

The competent cell of *B. subtilis* 168 prepared by the previous method (Kim et al., 2015) was transformed with SpCas9-carrying plasmid pHCas9 to construct BS-C100. Next, BS-C100 was transformed with the pAD123 derivatives containing donor DNA template and sgRNA transcription module. Half of the transformation mixture was spread on LB agar plate containing chloramphenicol (5 μg/ml) and neomycin (10 μg/ml). The other half was inoculated into same conditioned broth media with final 1% concentration for prolonged incubation. After culturing for different time periods, the cultured cells were serially diluted and spread on LB agar plate containing chloramphenicol (5 μg/ml) and neomycin (10 μg/ml). The mutations were confirmed by PCR, phenotypic screening, flow cytometry, and DNA sequencing.

### Flow Cytometry

Flow cytometry and data analysis were conducted using a Guava^®^ easyCyte cytometer (Millipore Corporation, Billerica, MA, United States). A Guava InCyte assay demonstrated the GFP activity of *B. subtilis* 168 and BS-C111 cells. *Bacillus* strains were pre-cultured overnight and main-cultured the next day in LB medium. All the samples were washed with phosphate-buffered saline (PBS) three times and resuspended in 1 ml of PBS for further flow cytometry analysis. Each sample went through 15000 cell counts for 600 s, and was mixed for 3 s at a medium rate.

## Results

### Determination of a Donor DNA Size for Homologous Directed Repair (HDR)

It has been established that 70 bp were required for RecE-dependent homologous recombination in *Bacillus* (Khasanov et al., 1992). In genome engineering using *mazF* counter-selectable marker, 125 bp-directed repeats for *in vivo* recombination were used to generate *bpr* deletion mutant (Zhang et al., 2006). To determine the size of the donor DNA template for HDR-mediated CRISPR-Cas9 in *B. subtilis*, we targeted the *spo0A* gene. Spo0A is a transcription regulator for initiation of sporulation in *B. subtilis*. Inactivation of *spo0A* prevents endospore formation and alters *Bacillus* cell morphology leading to cell lysis (Hamon and Lazazzera, 2001). Using two plasmids: pHCas9 and pB0A, we determined the appropriate size of the donor DNA for CRISPR-Cas9-mediated genome engineering. SpCas9-expressing plasmid pHCas9 (**Figure 1A**) was transferred into wild type *B. subtilis* 168 to give strain BS-C100. Prior to introducing the donor DNA template plasmid pB0A (**Figure 1B**), we transfer p0AgR, which contains only a *spo0A*-targeting sgRNA transcribing module, into BS-C100. Introduction of p0AgR produced no colonies on LB chloramphenicol (5 μg/ml) and neomycin (10 μg/ml) agar plate (data not shown). This result indicates that p0AgR was unable to generate the *spo0A* deletion mutant without a donor DNA template for homologous recombination.

To determine the minimum size of donor DNA template that retains high recombination efficiency we truncated the donor DNA template from 1 kb to 50 bp for each upstream and downstream (**Figure 2A**). The truncated donor DNA templates were cloned into *spo0A*-targeting sgRNA producing plasmids and produced pB0A-2, pB0A-1, pB0A-800, pB0A-400, pB0A-200, and pB0A-100. Introduction of each plasmids into BS-C100 resulted in strains containing a 32 bp deletion mutation in *spo0A*: BS-C101, BS-C102, BS-C103, BS-C104 BS-C105, and BS-C106. With a 2 kb donor DNA template (1 kb each from the N- and C-termini), mutation efficiency was 100%. However, with truncated donor DNA templates, from 1 kb to 400 bp, the mutation efficiencies were slightly lower as 91% on average (**Figure 2B**). With a 200 bp donor DNA template, there was a substantial decrease in mutation efficiency, which indicates that the donor DNA template should be longer than 400 bp for maintaining the high mutation efficiency using HDR-mediated CRISPR-Cas9 in *B. subtilis*. We also observed that mutation efficiency was very low without the pHCas9, as 8.7% even with a 2 kb donor DNA template. This suggests that the mutation generation strongly depends on SpCas9 (**Figure 2B**). The *spo0A* mutants were distinguished by morphological change (**Figure 2A**) and confirmed with DNA sequencing.

### Correlation between Deletion Size and Mutation Efficiency

To determine whether the size of the deletion region affects mutation efficiency in *B. subtilis*, we constructed 500 bp and 1 kb deletions in the *spo0A*. It has already been demonstrated that the oligonucleotide-mediated HDR had 30 and 6.5% mutation efficiency in *Lactobacillus* with, respectively, 501 and 1,002 bp deletions on the *lacL* gene (Oh and van Pijkeren, 2014). We obtained strains BS-C107 and BS-C108 by introducing pB0A-d500 and pB0A-d1000 into BS-C100. The 500 bp deletion mutant strain BS-C107 showed 97% mutation efficiency and the 1 kb deletion mutant strain BS-C108 showed 88% mutation efficiency (**Figure 2C**). Unlike in *Lactobacillus*, there was no significant decrease in mutation efficiency among three different *spo0A* mutants, BS-C102, BS-C107, and BS-C108. This result indicates that plasmid delivered SpCas9/sgRNA, along with 500 bp donor DNA template maintains high mutation efficiency for the deletion up to 1 kb in *B. subtilis*.

### CRISPR-Cas9 Mediated Large Genomic Deletion

**FIGURE 3:**
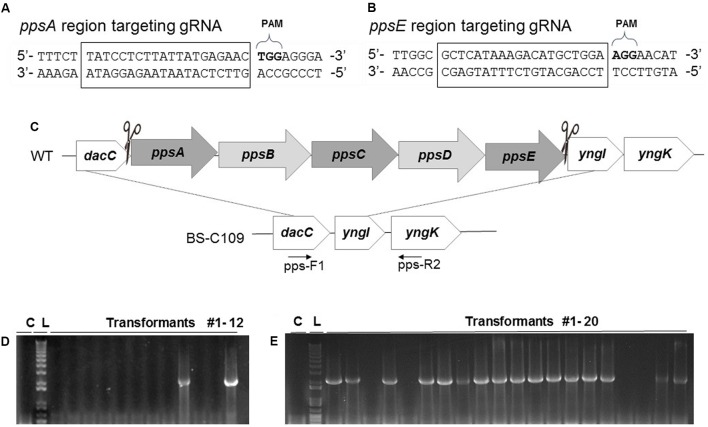
Construction of the *pps* operon (*ppsABCDE*) deletion mutation and its efficiency. **(A)** *ppsA* and **(B)** *ppsE*-targeting multiple gRNA sequences. All the squared 20 bp nucleotides were used as gRNA binding sites. **(C)** After DSBs occurred in *ppsA* and *ppsE*, double crossover at the *dacC* and *yngK* initiated to eliminate the *pps* operon (38 kb). Deletion was confirmed with PCR using two primers, pps-F1 and pps-R2. **(D)** Without prolonged incubation the *pps* operon deletion mutation was 16.7% efficient. **(E)** Prolonged incubation under selective pressure increased the mutation efficiency to 80%.

Previous large genomic deletion studies have reported low mutation efficiency (Jeong et al., 2015) or used prerequisite strains containing antibiotic resistance markers (Morimoto et al., 2009). To determine whether the CRISPR-Cas9 system can efficiently delete an operon-like large genomic region in the chromosome and leave it completely free of foreign DNA, we targeted the *pps* operon. Research using a minimal *B. subtilis* genome factory confirmed that the plipastatin-synthesizing 38 kb operon (*ppsABCDE*) is not essential for cell growth (Ara et al., 2007). First, we constructed a *ppsC* targeting sgRNA, which generates DSB on the middle of 38 kb *pps* operon. A donor DNA template, which contains the N- and C-termini of the *pps* operon was PCR amplified and cloned into the *ppsC* targeting sgRNA-generating plasmid. Then we introduced the resulting plasmid into pHCas9-containing BS-C100 and cultured on LB chloramphenicol (5 μg/ml) and neomycin (10 μg/ml) agar. However, the transformants failed to generate the entire operon deletion (data not shown). We suspected that the DSB site was generated too far from the each N-and C-termini recombination region to initiate an efficient double cross-over. Therefore, we designed two sgRNAs, which can produce DSBs at each end of *pps* operon near the HDR sites, to yield a precise large genomic deletion: one targeting the N-terminus of the *ppsA* and the other targeting C-terminus of the *ppsE* (**Figures 3A,B**). After complexing with SpCa9, *ppsA*- and *ppsE*-targeting sgRNA will generate DSBs on the N-and C-termini of the *pps* operon respectively.

We introduced pBP-dpps into BS-C100 and demonstrated whether the two DSBs, which completely eliminates the 38 kb in the *B. subtilis* chromosome, can yield an efficient mutagenic HDR (**Figure 3C**). The pBP-dpps transferred BS-C100 produced a strain BS-C109, and the newly obtained BS-C109 transformation reaction mixture was spread onto agar containing LB chloramphenicol (5 μg/ml) and neomycin (10 μg/ml). Transformation efficiency was 3.13 × 10^2^ transformants/μg DNA. To confirm deletion of the *pps* operon we used two primers, pps-F1 and pps-R2, and amplified 3′-*dacC* through 5′-*yngK*. In the wild type strain, this region is over 40 kb, which exceeds the maximum amplification size under our PCR condition. However, 38 kb of this 40 kb region is absent from the *ppsABCDE* deletion mutant and the remaining 2 kb can be amplified for the confirmation of the deletion mutation (**Figure 3C**). We obtained 217 transformants and 12 single colonies were randomly chosen for PCR amplification. Only 2 out of the 12 colonies (16.7%) were identified as *pps* operon deletion mutants (**Figure 3D**). The CRISPR-Cas9-mediated large genomic deletion achieved higher mutation efficiency than the synthetic gene circuit method (6.4%), but still had low mutation efficiency compared with the other CRISPR-Cas9-based mutations in *B. subtilis*.

### Iterative Genome Engineering for Maximizing the Mutation Efficiency

**FIGURE 4:**
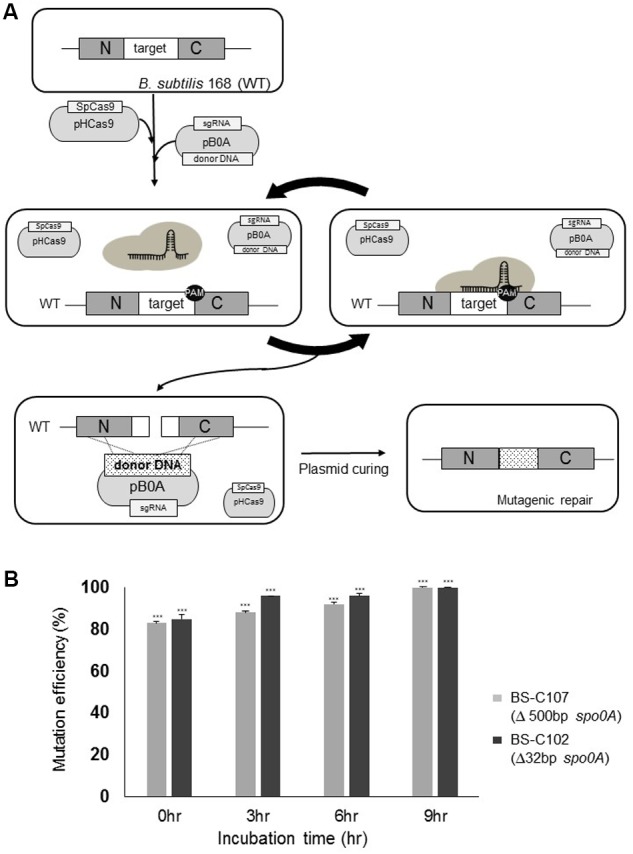
Iterative genome engineering system scheme. **(A)** Introduction of pHCas9 and pB0A into *B. subtilis* 168 allowed generation of *spo0A*-targeting sgRNA and SpCas9 complex. Prolonged incubation under the appropriate selection pressure allowed iterative DSB to occur on the *spo0A* target region until it becomes a desired mutant. **(B)** BS-C102 (black) and BS-C107 (gray) mutant strains were used to determine iterative genome engineering system under selective antibiotic conditions. Two strains showed an average mutation efficiency of 85% without prolonged incubation. Longer incubation resulted in a time-dependent increase in mutation efficiency, up to a maximum of 100%. Means of three independent technical replica experiments are presented; the error bars indicate standard deviations. ^∗∗∗^*P* < 0.001 compared with control without presence of pHCas9.

To increase the operon-like large genomic deletion efficiency, we developed an iterative system based on the plasmids used in this study. Two plasmids, which produce SpCas9, *spo0A*-targeting sgRNA, and donor DNA template, were stably maintained under selective pressure. For this reason, when the target chromosomal region escapes sgRNA/SpCas9 endonuclease activity, it will “iteratively” attack the target region to form DSBs until mutagenic repair occurs (**Figure 4A**).

To account for the iterative genome engineering system using CRISPR-Cas9, we spread half (100 μl) of the newly obtained BS-C109 transformation reaction mixture onto the agar containing LB chloramphenicol (5 μg/ml) and neomycin (10 μg/ml). The other half (100 μl) of the BS-C109 transformation mixture was cultured for 16 h on LB chloramphenicol (5 μg/ml) and neomycin (10 μg/ml) broth media. The mixture was then spread in the same agar medium. Without the prolonged incubation, only 16% of the transformants were confirmed as *pps* operon deletion mutants. Surprisingly, after prolonged incubation, 16 out of the 20 colonies (80%) were identified as *pps* operon deletion mutants (**Figure 3E**). This result provided an evidence that prolonged incubation increased the mutation efficiency.

To confirm the effect of prolonged incubation on increasing the mutation efficiency, we introduced pB0A-1 and pB0A-d500 into BS-C100 respectively, and obtained strains BS-C102 (32 bp *spo0A* deletion) and BS-C107 (500 bp *spo0A* deletion). Each transformation reaction mixture was time-series incubated under antibiotic pressure. Without prolonged incubation (0 h), BS-C107 and BS-C102 transformants showed average mutation efficiencies of 83 and 85% respectively. Incubation for longer periods resulted increase in mutation efficiencies up to 100% in time dependent manner on both strains (**Figure 4B**). These results demonstrated that the mutation efficiency can be maximized by maintaining SpCas9, sgRNA transcription module and donor DNA template containing plasmids under selective pressure.

### Point Mutation and Gene Insertion

**FIGURE 5:**
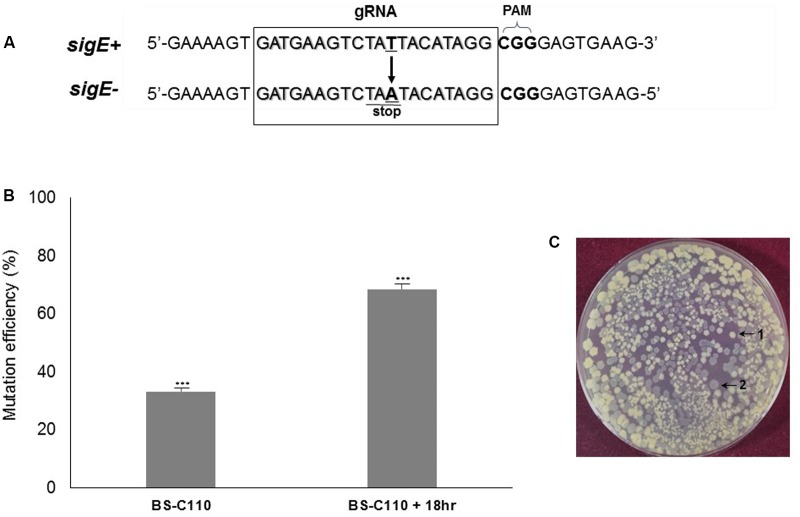
*sigE* point mutation efficiency. **(A)** The squared region represents the 20 bp *sigE*-targeting gRNA sequence. The thymine (T) was changed into adenine (A) to produce a stop codon. **(B)** Prolonged incubation (18 h) in selective media increased mutation efficiency from 33 to 68%. The means of three independent technical replica experiments are presented; error bars represent standard deviations. ^∗∗∗^*P* < 0.001 compared with control without presence of pHCas9. **(C)** Two different *B. subtilis* phenotypes; 1 indicates wild type *B. subtilis* 168 whereas 2 indicates transparent *sigE* mutant BS-C110.

We targeted *sigE* to determine whether our system can produce genomic point mutation efficiently. Mutation in *sigE* gene is known to inactivate the sporulation cascade causing morphological change in *B. subtilis* (Stragier et al., 1988). By changing thymine (T) into adenine (A), we created a stop codon (TAA) at the N-terminus of *sigE* to prevent its translation, which causing a significant morphological change in the cell (**Figure 5A**). Transfer of *sigE* point mutation-generating plasmid pBE-pm into BS-C100 resulted a strain BS-C110, and half of the fresh transformation reaction mixture (100 μl) was spread onto agar containing LB chloramphenicol (5 μg/ml) and neomycin (10 μg/ml). The initial transformation efficiency was 2.84 × 10^2^ transformants/μg DNA. On average, 33% of transformants (56 out of 169) showed a *sigE* mutant phenotype in this medium. Ten transparent colonies were randomly selected and sequence analysis confirmed that all 10 colonies contained *sigE* point mutation (T → A). We then cultured the other half of the transformation reaction mixture (100 μl) overnight in the same medium, under the same conditions. Spreading 100 μl of 10^-5^-diluted BS-C110 overnight culture produced an average of 805 colonies and about 68.5% of mutants were shown to be *sigE* mutants (**Figure 5B**). Among the transparent colonies (**Figure 5C**), 10 colonies were randomly selected for DNA sequencing. Sequence analysis confirmed that all 10 colonies contained *sigE* point mutation (T → A). This result indicates that given prolonged incubation helped to increase genomic point mutation efficiency.

**FIGURE 6:**
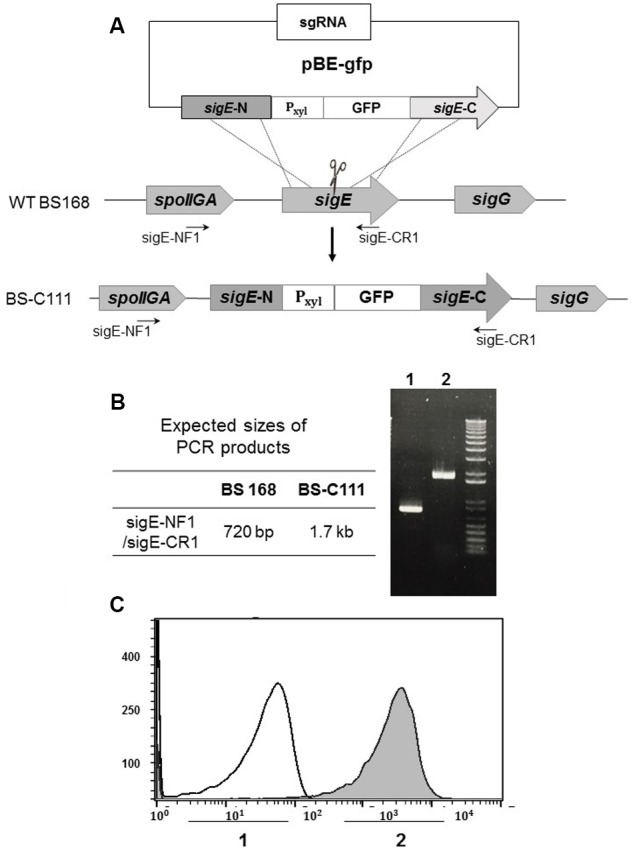
Construction of green fluorescence protein (GFP) insertion in *sigE*. **(A)** Along with pHCas9, pBE-gfp was used to deliver sgRNA and the donor DNA template to generate GFP insertion in the *sigE* gene through HDR. **(B)** Insertion of P_xyl_-gfp was confirmed with PCR and **(C)** Guava InCyte flow cytometry assay; 1 indicates the *B. subtilis* 168 control; 2 is pBE-gfp-transferred BS-C111.

As well as generating knock-out mutants, we determined the effect of our iterative genome engineering method on generating knock-in mutants in *B. subtilis*. We designed pBE-gfp, which generates a GFP insertion mutant on the *sigE* gene. Introducing pBE-gfp into pHCas9-containing BS-C100 resulted in BS-C111 strain (**Figure 6A**). We spread half (100 μl) of newly obtained BS-C111 transformation reaction mixture onto LB agar containing 1% xylose in addition to chloramphenicol (5 μg/ml) and neomycin (10 μg/ml), and the transformation efficiency was 2.04 × 10^2^ transformants/μg DNA. Among 202 single colonies, 135 colonies (66.8%) had a *sigE* mutant phenotypes with GFP expression. The other half (100 μl) of transformation reaction mixture was cultured overnight in the same medium at 37°C 200 rpm. As a control, *B. subtilis* 168 was cultured in LB without any antibiotics. Spreading 100 μl of 10^-4^-diluted BS-C111 overnight culture yielded 442 distinct colonies of which 428 *sigE* mutants showed GFP expression (96.8%). Six of the 428 colonies were randomly selected for PCR amplification with primers sigE-NF1 and sigE-CR1. We verified the insertion of a P_xyl_-GFP cassette using PCR and the *sigE* mutant-specific morphological change (**Figure 6B**) as well as DNA sequencing. Guava InCyte assay confirmed the GFP expression of BS-C111, while no GFP expression observed with the wild type strain *B. subtilis* 168 (**Figure 6C**). The iterative genome engineering method with prolonged incubation under selective pressure had also confirmed its ability to increase the insertion mutation efficiency.

### Plasmid Curing

For industrial and food-grade applications, traces of foreign DNA in the cells must be removed by plasmid curing. Previous research had demonstrated that the *B. subtilis* integration vector pHT01 and expression vector pAD123 have 50 and 43% stability under overnight antibiotic-free conditions, respectively (Yamashiro et al., 2011). To confirm the curing efficiency of plasmid pHT01 derivative pHCas9 containing a neomycin-resistance gene, BS-C101 was cultured overnight in 1 ml LB chloramphenicol (5 μg/ml) broth medium without neomycin. Single colonies were isolated by serial dilution and 70 colonies were randomly duplicated on agar containing LB chloramphenicol and LB neomycin. Sixteen out of 70 colonies did not grow on LB neomycin-containing plates, indicating that 22.8% of the Cas9-containing pHT01 derivative pHCas9 had been removed. One of the 16 colonies from the LB chloramphenicol-containing replica plate was cultured in 1 ml of LB medium to determine the curing efficiency of the sgRNA and donor DNA template-containing pAD123 derivative, pB0A. Duplicate samples of 70 randomly selected colonies were transferred onto agar plates with or without LB chloramphenicol (5 μg/ml). Thirty-five out of 70 colonies were not able to grow on agar containing LB chloramphenicol (5 μg/ml), indicating that both plasmids were easily cured by serial culture in antibiotic-free conditions (data not shown).

## Discussion

By considering the low efficiency on generating *pps* operon deletion (6.4%) in *B. subtilis* using a synthetic gene circuit (Jeong et al., 2015); introducing the CRISPR-Cas9 system gave more powerful precision on generating operon-like large genomic deletion without leaving a foreign DNA trace in *B. subtilis*. We speculated that the dramatic increase in efficiency, from 16 to 80%, of the *pps* operon deletion was due to prolonged incubation in selective media. Since SpCas9, target-specific sgRNA, and donor DNA template were delivered in plasmids, constant formation of SpCas9/sgRNA endonuclease complex was thought for the iterative target binding on the chromosome. It seems likely that the DSBs were created continuously on the target chromosomal region until mutagenic repair occurs that no longer contains SpCas9/sgRNA binding site. This may have increased the efficiency of large genomic deletion in *B. subtilis*. Also, unlike previous attempts at CRISPR-Cas9-mediated mutagenesis in *B. subtilis*, the iterative system that we developed in this study had high mutation efficiencies – up to 100% – for other site-directed mutations including base deletion, point mutation and gene insertion, without any additional methods.

Plasmid-based CRISPR-Cas9 systems are reported to generate more off-target effects than the mRNA or protein forms of SpCas9 due to their high stability in eukaryotes (Kim et al., 2014). SpCas9 endonuclease can generate DSBs when sgRNA binds to a non-target site that has high sequence homology between 16 and 19 bp. However, off-target effects are less common in CRISPR-based genome engineering in bacterial species as they have smaller genomes than eukaryotes (Zhang et al., 2016). We assessed off-target effects in *B. subtilis* by introducing p0AgR, a plasmid containing only a *spo0A*-targeting sgRNA-transcribing module but no repair template. After transfer of p0AgR into pHCas9 containing BS-C100, no colonies were observed. This result indicates that CRISPR-Cas9-mediated genome engineering in *B. subtilis* is strongly dependent upon HDR. Many prokaryotes respond to CRISPR-Cas9-based DSBs by homologous region-specific HDR because NHEJ mechanism is impaired during DNA replication (Ayora et al., 2011).

After demonstration of heterologous expression of SpCas9 (Sapranauskas et al., 2011), the CRISPR-Cas9 was developed in many prokaryotic species, including *B. subtilis*, as a promising foreign DNA-free genome engineering technique. Despite the simplicity of CRISPR-Cas9-mediated prokaryotic genome engineering, previous studies also used recombinase or counter-selectable markers in order to increase mutation efficiency. Lambda-Red (λ-Red) recombineering system-assisted CRISPR-Cas9 in *E. coli* produced a deletion mutation on *cadA* gene with 86% efficiency and up to 100% efficiency was achieved for a deletion mutation on *tkrA* of *Tatumella citrea*. However, the authors reported that mutation efficiency was significantly lower in the absence of Red recombinase (Jiang et al., 2015). Since the iterative system does not require preparation of prerequisite strains containing recombinases or counter-selectable markers, nor insertion of an antibiotic resistance marker at the secondary region on the chromosome, it gratifies the food-grade status of *B. subtilis* and maintain the simplicity of the CRISPR-Cas9 to generate desired mutant quickly in relative to the previous engineering methods. This indicates that our CRISPR-Cas9-mediated iterative genome engineering system can generate precise mutations in difficult-to-transform prokaryotic strains and may lead to advances in genome reduction, food-grade applications, industrial fermentations, and bioremediations.

## Author Contributions

YS, S-YP, J-GP, and S-KC designed the experiment. YS, S-YP, and E-HP realized all experiments. S-HP, E-JK, J-GP, and S-KC participated in coordination and discussion of the study. YS, S-YP, and S-KC wrote the paper. All authors reviewed the manuscript.

## Conflict of Interest Statement

The authors declare that the research was conducted in the absence of any commercial or financial relationships that could be construed as a potential conflict of interest.
